# Auto-Continuous Synthesis of Robust and Hydrophobic Silica Aerogel Microspheres from Low-Cost Aqueous Sodium Silicate for Fast Dynamic Organics Removal

**DOI:** 10.3390/gels8120778

**Published:** 2022-11-28

**Authors:** Ziqian Sun, Zhiyang Zhao, Yong Kong, Jian Ren, Xing Jiang, Xiaodong Shen

**Affiliations:** 1 College of Materials Science and Engineering, Nanjing Tech University, Nanjing 210009, China; 2 State Key Laboratory of Materials-Oriented Chemical Engineering, Nanjing 210009, China

**Keywords:** silica aerogel, microsphere, hydrophobic, polymer cross-linking, adsorption

## Abstract

An efficient auto-continuous globing process was developed with a self-built apparatus to synthesize pure silica aerogel microspheres (PSAMs) using sodium silicate as a precursor and water as a solvent. A hydrophobic silica aerogel microsphere (HSAM) was obtained by methyl grafting. A reinforced silica aerogel microsphere (RSAM) was prepared by polymer cross-linking on the framework of the silica gel. The pH value of the reaction system and the temperature of the coagulating bath were critical to form perfect SAMs with a diameter of 3.0 ± 0.2 mm. The grafted methyl groups are thermally stable up to 400 °C. Polymer cross-linking increased the strength significantly, owing to the polymer coating on the framework of silica aerogel. The pore volumes of HSAM (6.44 cm^3^/g) and RSAM (3.17 cm^3^/g) were much higher than their state-of-the-art counterparts. Their specific surface areas were also at a high level. The HSAM and RSAM showed high organic sorption capacities, i.e., 17.9 g/g of pump oil, 11.8 g/g of hexane, and 22.2 mg/g of 10 mg/L methyl orange. The novel preparation method was facile, cost-effective, safe, and eco-friendly, and the resulting SAM sorbents were exceptional in capacity, dynamics, regenerability, and stability.

## 1. Introduction

Silica aerogels have large specific surface areas, nanopore volumes, and porosities and show excellent performances in thermal insulation, adsorption, catalysis, and many other aspects [1,2,3,4,5,6]. Recently, it has caused wide public concern to remove organic contaminants from water with hydrophobic silica aerogels [7,8]. Tetraethoxysilane (TEOS) and tetramethoxysilane (TMOS) are generally used as precursors to synthesize silica aerogels, which have a lot of hydroxy groups that can be grafted with methylic silanes to obtain hydrophobic silica aerogels [9,10,11]. The methyl groups of hydrophobic silica aerogels can enhance the interaction between silica aerogel and organic contaminants, improve the sorption selectivity and capacity of the organics, and avoid network failure from water. Qin et al. synthesized hydrophobic silica aerogel powder by modifying TEOS-derived silica gel with trimethylchlorosilane (TMCS) [12]. The sample with 12 h modification had a specific surface area of 875 m^2^/g, a pore volume of 0.56 cm^3^/g, and a phenol absorption capacity of 142 mg/g from water. Sert Çok et al. investigated the effect of a hydrophobic modifier on the structure and organic sorption performance of hydrophobic silica aerogel monoliths [13]. TMCS was the optimal modifier, and the corresponding hydrophobic silica aerogel had a specific surface area and a pore volume of 789 m^2^/g and 2.49 cm^3^/g, respectively. The hexane and vegetable oil absorption capacities were 7.0 and 8.5 g/g, respectively, and were stable within six cycles.

The use of silicon alkoxide is efficient for synthesizing hydrophobic silica aerogels for water treatment. However, the organic precursors are expensive, which limits the engineering application of silica aerogels. As an alternative, sodium silicate is low-cost and has a broad resource of raw material. Instead of organic solvents, such as ethanol, for silicon alkoxide-based silica aerogels, water is used as the solvent for the sodium silicate-based silica aerogels, which is safer and greener [14]. The sodium silicate-derived silica aerogels with TMCS as a hydrophobic modifier achieved a specific surface area of 870 m^2^/g and a pore volume of 4.27 cm^3^/g, which were as high as those of the silicon alkoxide-based counterparts [15,16,17,18,19]. The sodium silicate-based hydrophobic silica aerogel monolith with a specific surface area of 870 m^2^/g and a pore volume of 4.0 cm^3^/g achieved a stable heptane absorption capacity (2.97 g/g) within 10 cycles [19]. The sodium silicate-based hydrophobic silica aerogel powder with a specific surface area of 246 m^2^/g had a silicone oil absorption capacity of 1.32 g/g [20].

The aforementioned studies indicate that current products are mostly monoliths and powders, which are difficult to use in practical projects. The aerogel powders used in flow through separation systems could lead to high backpressure and low permeability [21]. The monoliths are adverse to mass transfer, leading to low sorption capacity owing to the short-circuiting effect. Microsphere is a new product type of aerogels, which was synthesized by the ball drop method, emulsion method, and jet cutting method, which are potential in water treatment [22,23,24,25,26,27,28,29]. The microsphere avoids the blockage of the fixed adsorbent bed and offers good interaction between adsorbent and adsorbate [21,30,31]. Moreover, microspheres are easy to recycle from water for regeneration. The sodium silicate-based hydrophobic silica aerogel microsphere has also been successfully synthesized [32]. Based on the ball drop method, the silica gel microsphere was formed by slowly dropping the silica sol into the silicon oil bath, the silica gel microsphere was modified with TMCS and dried with ambient pressure drying (APD) to form the hydrophobic silica aerogel microsphere with a specific surface area of 515 m^2^/g and a pore volume of 1.48 cm^3^/g. However, the Rhodamine B adsorption performance of the hydrophobic silica aerogel microsphere was much lower than its hydrophilic counterparts.

The sodium silicate-based aerogel microsphere is a promising sorbent for organic removal from water. However, the inherent fragility of silica aerogels still restricts their engineering application in water treatment. Moreover, the intermittent ball drop method for molding microsphere is inefficient. Hence, a series of silica aerogel microspheres (PSAM, HSAM, and RSAM) were synthesized from sodium silicate via a continuous globing processing on a self-built apparatus in this work. The hydrophobic silica aerogel microsphere (HSAM) with large pore volumes and specific surface areas was synthesized by grafting a hydrophobic modifier onto the pure silica aerogel microsphere (PSAM). To overcome the fragility of the HSAM, the reinforced silica aerogel microsphere (RSAM) was synthesized by the cross-linking of polymers onto the PSAM. The synthesis mechanisms, structures, and organic sorption performances of these SAMs were investigated.

## 2. Results and Discussion

### 2.1. Auto-Continuous Globing Process and Reaction Mechanism

**Figure 1:**
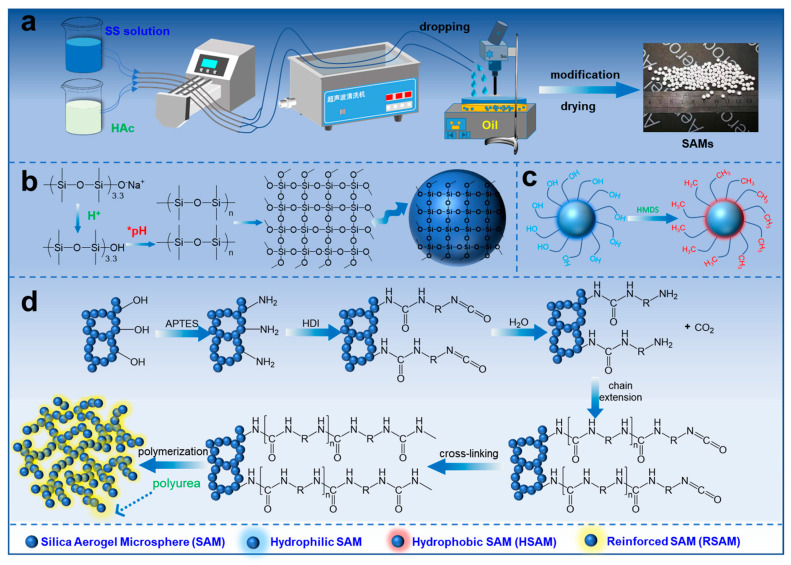
(**a**) The auto-continuous globing process of SAMs and (**b**–**d**) the schematic of the sphericalization, hydrophobization, and reinforcement.

Figure 1 and Video S1 vividly show the schematic and the auto-continuous globing process of SAMs. All the silica aerogels go through a sol–gel process to form a wet gel. However, the gelation mechanism of the microspheres is different from the monoliths and powders. As shown in Figure 1a, to obtain the perfect gel microsphere, the gelation kinetics of the solution or sol should not be fast before dropping and very fast in the coagulating bath. Therefore, the ASS and HAc solutions were prepared separately, and the dropping was performed as soon as the solutions were a mixture in the silicone tube. The mixing was carried out at room temperature to reduce the reaction kinetics, and the gelation was performed in the silicon oil bath at 90 °C to improve the reaction kinetics. The mixture and dimethicone are not mutually soluble, which leads to the formation of spherical droplets. In any event, the pH value is dominant. To form the perfect SGM, the optimal pH value is 5.74 ± 0.05, which leads to a gelation time of 4 ± 1 min at room temperature and less than 5 s at 90 °C. A pH volume beyond 5.74 ± 0.05 led to a longer gelation time in the silicon oil bath, which resulted in the serious aggregation of the liquid droplets. Moreover, the temperature of the coagulating bath should be high enough to accelerate the formation of SGM. The lower temperature of the silicon oil bath (70 °C) led to the mild aggregation of spherical droplets. The sol–gel process of the organic alkoxides to form silica gels depends on the hydrolysis and dehydration condensation of the silica alkoxides. The sol–gel behavior of the sodium silicate is similar to the silicon alkoxides.

As shown in Figure 1b, sodium silicate was firstly protonated to form the Si-OH species under acid condition, then the Si-OH groups condensed via dehydration to form Si-O-Si linkages, generating silica colloidal particles. Finally, the silica gel was formed through the aggregation of silica colloidal particles. The PSAM could be obtained after the drying of the gel. The formation of HSAMs is attributed to the graft of -CH_3_ species on the framework of the aerogel.

As shown in Figure 1c, the functional precursor, HMDS, was grafted onto the framework of the silica gel based on the formation of the Si-O-Si-(CH_3_)_3_ species. The non-polar -CH_3_ groups in the HSAM endow them with hydrophobicity.

The synthesis mechanism of the RSAM is presented in Figure 1d. The SGM was firstly functionalized with amino groups (-NH_2_) by grafting the APTES onto the framework of the SGM based on the dehydration reaction between the surface hydroxyl of SGM and the hydrolyzed species of APTES. HDI was grafted onto the framework of the SGM by reacting with the amino groups. Along with the HDI molecules that reacted with the amino groups one by one, the polymer chains were formed on the framework of the SGM; thus, the brittle silica framework was coated with robust polyurea chains [33,34]. As a result, the SAM was reinforced by polymer cross-linking.

### 2.2. Chemical Component

**Figure 2:**
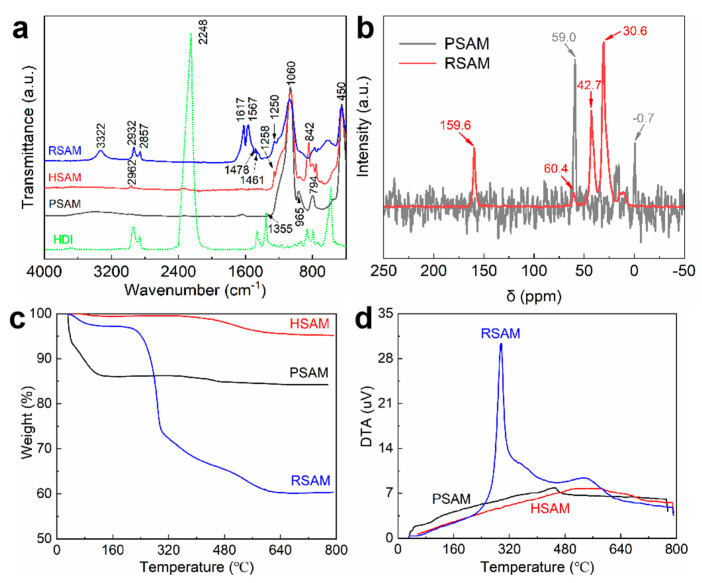
(**a**) FTIR spectra of SAMs and HDI. (**b**) Comparative ^13^C NMR characterization of the PSAM and RSAM. (**c**,**d**) TG and DTA curves of SAMs.

The FTIR spectra of the HDI, PSAM, HSAM and RSAM are shown in Figure 2a. All the SAM samples have three bands around 450, 794, and 1060 cm^−1^, which are attributed to the deformation vibration of O-Si-O, the symmetric stretching vibration of Si-O (Si-O-Si), and the antisymmetric stretching vibration of Si-O-Si, respectively [35]. The bands around 950–965 cm^−1^ are assigned to the Si-O in-plane stretching vibration of the Si-OH species [35]. The Si-OH species gradually disappeared from PSAM to HSAM and RSAM. This reveals that the Si-OH groups in the SGM were consumed by grafting HMDS and APTES molecules, as illustrated in Figure 2a. For the HSAM, the presence of the C-H stretching vibration of -CH_3_ at 2962 cm^−1^, the C-H bending of -CH_3_ at 1258 cm^−1^, and the Si-C stretching vibration of Si-CH_3_ at 842 cm^−1^ suggest that the hydrophobic Si-CH_3_ species of HMDS were successfully grafted [35,36]. For the RSAM, the C-H vibration bands at 2932, 2857, 1250, 1478, and 1461 cm^−1^ are attributed to the methylene groups, demonstrating the loading of HDI and APTES moieties on the RSAM [35]. The band at 3322 cm^−1^ of the RSAM results from the N-H stretching vibration of polyurea and APTES moieties [37]. The bands of HDI at 2248 and 1355 cm^−1^ are attributed to the antisymmetric stretching vibration and symmetric stretching vibration of the N=C=O groups of HDI [38]. These bands do not appear in the spectrum of the RSAM, indicating that the HDI monomer was loaded on the RSAM via polymerization to form polyurea structures, and the polymer cross-linking of the SAM was achieved. The bend around 623 cm^−1^ of the RSAM is mainly attributed to the bending vibration of O=C=N in HDI, indicating the presence of less incompletely reacted HDI. Correspondingly, the C=O and C-N-H stretching vibrations of the polyurea species were observed around 1618 and 1567 cm^−1^, respectively. The SEM mapping confirms HDI loading on the RSAM (Figure S1).

Figure 2b shows the comparative ^13^C NMR characterization of the pure silica aerogel microsphere (PSAM) and the HDI-reinforced silica aerogel microsphere (RSAM). As shown in the figure, the new vibration peak at around 159.6 ppm is assigned to the newly formed amino groups, indicating that HDI reacted with the -NH_2_ groups of silica clusters forming the -NH-C-O-NH- chains [39]. The intense peaks at 42.7 and 30.6 ppm are attributed to the -CH_2_ groups of the HDI cross-linker [40,41]. The peak at around 60 ppm induced by ethoxylation is evident in the PSAM [42], while a very weak peak can be observed in the RSAM, further verifying the polymerization between -NH_2_ and -NCO groups. The above results suggest the existence of -NCO groups, indicating the successful cross-linking of HDI.

### 2.3. Thermal Performance

TG and DTA curves of SAMs are shown in Figure 2c,d. The weight loss below 100 °C derives from the adsorbed water. The SAM presents more weight loss of water than the HSAM and RSAM as it possesses abundant hydroxyl species, which could be consumed via HMDS and APTES grafting. The hydroxyl groups readily adsorb moisture via hydrogen bonding. The slight weight loss of the PSAM above 100 °C is from the elimination of bonded water and hydroxyl species, which suggests that the dehydration reaction between the two Si-OH groups were carried out adequately, and the dominant species of the PSAM is silica. There was no obvious weight loss between 100 and 400 °C for HSAM, indicating that the grafted Si-CH_3_ species have excellent standing stability below 400 °C. The thermal stability of the hydrophobic groups of the HSAM is better than its state-of-the-art counterparts, which generally decomposed below 350 °C. The weight loss of the -CH_3_ species from 400 to 800 °C was very small, revealing that the HSAM has respectable thermal stability at an elevated temperature. The weight loss above 250 °C of the RSAM corresponds to the decomposition of the polyurea derived from the polymerization of HDI moieties. The DTA curve of the RSAM confirms that the polyurea in the RSAM starts to decompose at 250 °C and rapidly decomposes around 300 °C. The considerable weight loss of the RSAM above 250 °C suggests the effective cross-linking of HDI.

### 2.4. Microstructure and Morphology

**Figure 3:**
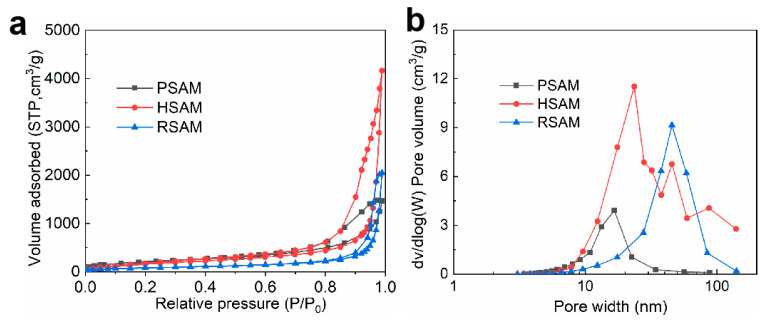
(**a**) N_2_ adsorption/desorption isotherms of SAMs. (**b**) Pore size distribution curves of SAMs.

**Figure 4:**
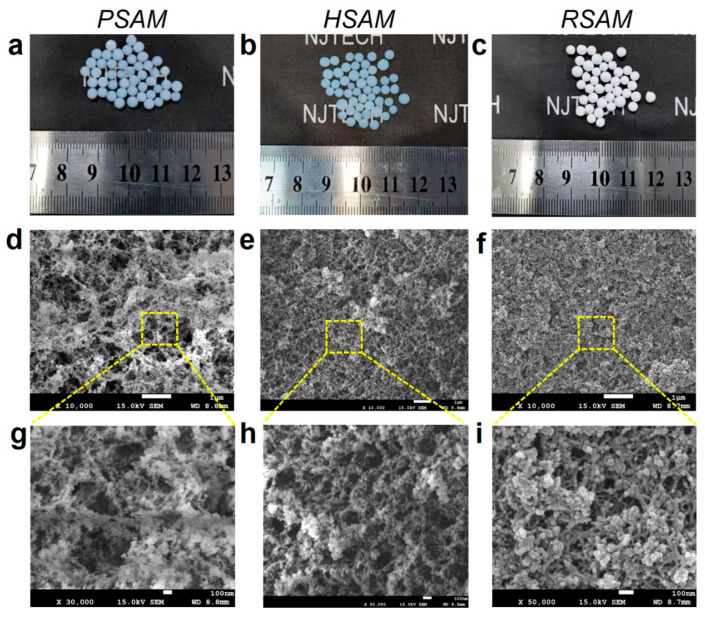
Optical images and SEM images of SAMs: (**a**–**c**) Optical images of the PSAM, HSAM, and RSAM. (**d**–**f**) SEM images of the PSAM, HSAM, and RSAM with low magnification. (**g**–**i**) High magnification.

The larger the ratio of the solvent to silicon source, the larger the specific surface area and pore volume of the aerogel obtained but the poorer the mechanical properties. The practical value of SAMs was taken into account, and a ratio was chosen that would ensure good mechanical properties of the PASM and HSAM with the best possible specific surface area and pore volume. A N_2_ adsorption test was performed to comprehensively understand the microstructure of SAMs. The N_2_ adsorption/desorption isotherms and pore size distribution curves are shown in Figure 3a,b. All the samples show type IV isotherms with H2 hysteresis loops, indicating that SAMs have a mesoporous structure constructed by irregular spherical pores and nanoparticles. The absence of the saturation adsorption platform close to 1 of P/P_0_ indicates the existence of macropores and large voids in the SAMs, which can be observed in the pore size distribution curves. It can be found from the pore size distribution curve presented in Figure 3b and pore structure data presented in Table 1 that the pore size and pore volume of the HSAM and RSAM are distinctly larger than that of the PSAM, which seems incompatible with common sense. The PSAM possesses the lowest apparent density at 0.080 g/cm^3^; its total pore volume is about 12 cm^3^/g, in theory, considering the skeleton density of SiO_2_ (2.2 g/cm^3^). However, the measured pore volume (2.27 cm^3^/g) is much lower than the theoretical pore volume, which means there are many pores that cannot be detected by the N_2_ adsorption method. As observed in the SEM image of PSAM, the pore width of these large voids is mostly larger than 100 nm, which is beyond the detect region of the N_2_ adsorption method. Generally, the N_2_ adsorption method only detects pores of 1–100 nm normally. The large voids are partially filled after surface modification with HMDS; thus, most pores of the HSAM are nanoscale, as presented in Figure 4e,h. The nanopore volume of the HSAM (6.44 cm^3^/g) is much higher than its state-of-the-art counterparts, but the specific surface area (652 m^2^/g) is mediocre [12,13,14,15,16,17,18,19]. The measured pore volume and specific surface area of the RSAM substantially decrease relative to the HSAM, which could be explained by the nanopores that were blocked by polymer cross-linking. Nonetheless, the specific surface area and pore volume of the RSAM are higher than its state-of-the-art counterparts [33,34,43]. As seen from the pore size distribution, the RSAM has relatively large pores, as the smaller pores are blocked after APTES grafting and polyurea coating, only leaving the larger pores. Overall, the porosity and total pore volume of the aerogel depend on the apparent and skeleton densities. However, the measured pore volume and specific surface area are closely linked to the pore size.

The photographs and SEM images of SAMs are shown in Figure 4. All SAMs exhibit a high degree of sphericity. The PSAM and HSAM are translucent, while the PSAM is white and opaque. The diameter of SAMs is 3.0 ± 0.2 mm. The apparent densities of the PSAM, HSAM, and RSAM are 0.08, 0.11, and 0.166 g/cm^3^, respectively. The dramatic increase in the apparent density from the PSAM to the HSAM and RSAM demonstrates that plenty of Si-CH_3_ and polyurea species are grafted on the HSAM and RSAM, respectively. The SEM images demonstrate that the PSAM and HSAM showed a nanoporous network constructed by colloidal particles and nanopores, which is the same as the typical silica aerogels [3,44,45]. The graft of the Si-CH_3_ species leads to the decrease in large voids, and the microscopic morphology of the HSAM is similar with the reported hydrophobic silica aerogels [9,11,46,47]. However, the RSAM with polymer cross-linking showed a network with agglomerations, of which the particles were connected to each other as HDI-derived polyurea coated on the framework [33]. The TEM images confirmed the naked framework of the PSAM and the polymer coated framework of the RSAM (Figure S4). The results well confirmed the successful reinforcement of the RSAM.

### 2.5. Mechanical and Hydrophobic Performance

**Figure 5:**
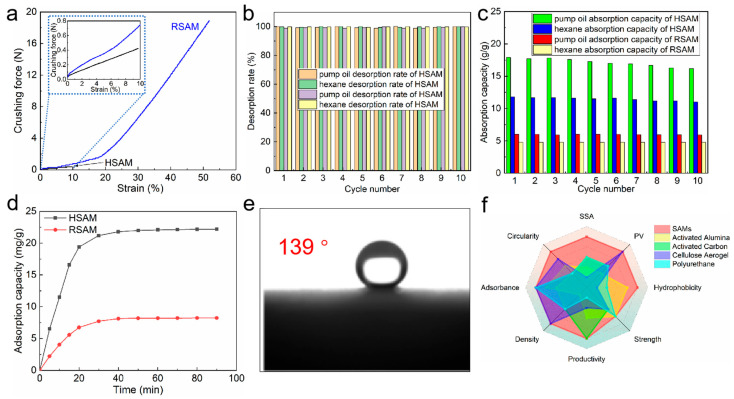
(**a**) Crushing force–strain curves of the HSAM and RSAM. (**b**) Desorption rates of the HSAM and RSAM. (**c**) Cyclic absorption capacities of the HSAM and RSAM. (**d**) MO adsorption kinetics of the HSAM and RSAM (0.10 ± 0.01 g of sorbent, 10 mg/L MO solution, and 100 and 250 mL MO solution for the HSAM and RSAM, respectively). (**e**) Water contact angle of the HSAM. (**f**) Qualitative comparison of typical properties of commercial adsorption materials, PSAM, HSAM, and RSAM.

Hydrophobic silica aerogels are commonly used for organics’ removal from water and have shown good performances. The low compressive strength limits the application of hydrophobic silica aerogels significantly. To demonstrate the enhanced compressive strength of the RSAM, 10 microspheres with a diameter of 3 ± 0.2 mm were used to conduct the compression test (Figure S5). The crushing force–stress curves of the HSAM and RSAM are shown in Figure 5a. Polymer cross-linking can improve the strength of the SAM significantly. The crushing force increases from 0.4 N for the HSAM to 19.2 N for the RSAM. It is understandable when the microstructure of the HSAM and RSAM is taken into consideration. The nanoparticles that construct the framework of the RSAM are coated with polyurea, which improve the strength of the framework.

After HMDS modification, the HSAM exhibited a water contact angle of 139°, as seen in Figure 5e. To confirm the thermal stability of the HSAM observed from the TG curve, the samples were treated in a muffle furnace. The results demonstrate that the HSAM can retain hydrophobicity at an elevated temperature up to 400 °C in Figure S3. Although the RSAM is hydrophilic and has a water contact angle of 73°, as seen in Figure S2, the polymer coating can avoid damage from water to a large extent, which is very different from the PSAM (Videos S2 and S3). The network of the PSAM cracked when contacting water, and the PSAM was destroyed by water. However, the PSAM’s macroscopic structure was retained in the water. This reveals that the RSAM can be used for water treatment, although it is not hydrophobic.

### 2.6. Adsorption Performance and Cyclic Stability

The saturated absorption capacities of the HSAM and PSAM for pump oil and hexane are summarized in Table 2. The HSAM possesses better oil absorption performance than the RSAM, owing to its large pore volume and specific surface area. Moreover, the methyl species in the HSAM favor the interaction between HSAM and oils. The oil absorption capacities of the HSAM and RSAM are as high as its state-of-the-art counterparts [12,13,19,20,46]. The regenerability is significant for the sorbent, which can reduce the long-running cost. The desorption rates and cyclic absorption capacities of the HSAM and RSAM within 10 cycles are shown in Figure 5b,c. Both the HSAM and RSAM can be regenerated effectively; the desorption rates are close to 100%. Although the RSAM has lower absorption capacities, owing to the small pore volume and specific surface, it shows better cyclic stability due to the reinforced network. The absorption capacities of the HSAM gradually decrease along with the cycle.

Except oil, dyestuff is another contaminant for water. MO was used to estimate the dyestuff adsorption performance of SAMs. The MO adsorption kinetics of SAMs are shown in Figure 5d. It should be noted that a low MO concentration and restricted MO solution are employed to evaluate the MO adsorption performance, which is challenging for a sorbent. The HSAM and RSAM achieve adsorption equilibrium within 40 min, and the adsorption halftime is only 9.6 and 10.5 min for the HSAM and RSAM, respectively [48,49]. This reveals that the HSAM and RSAM are dynamic for diluting MO adsorption from water, and the kinetics are not affected by the pore structure and chemical constituent. The MO concentration should be the dominant factor for adsorption dynamics. The MO adsorption capacity of the HSAM is much higher than that of the RSAM, which is understandable when the pore volume and specific surface area are taken into consideration. The equilibrated MO adsorption capacities of the HSAM and RSAM are 22.2 and 8.24 mg/g, respectively, which are much better than that of the reported hydrophobic silica aerogel microspheres, monoliths, and powders [32]. Figure 5f provides a qualitative comparison of SAMs with materials such as cellulose aerogels, activated carbon, activated alumina, and polyurethane. Compared with other adsorbent materials reported in the literature and conventional commercial adsorbent materials [50,51,52,53,54,55,56], as concluded in Table 3, SAMs have a more comprehensive, excellent performance in terms of both microstructure, such as specific surface aera (SSA), and pore volume (PV), and practical performance, such as hydrophobicity, strength, adsorption capacity, cycling performance, and productivity.

## 3. Conclusions

SAMs can be synthesized via a continuous globing process on a self-built apparatus with sodium silicate as a precursor and water as a solvent. To obtain the perfect SAM, the pH value of the reaction system and the temperature of the coagulating bath are critical. The preferred pH value of the reaction solution and the temperature of the dimethicone bath are 5.74 ± 0.05 and 90 °C, respectively. The hydrophobic modification to obtain the HSAM can be achieved by grafting Si-CH_3_ species on the SAM. The chemically bonded methyl groups show a surprising thermal stability up to 400 °C. The reinforcement of the SAM to form the RSAM was carried out based on polymer cross-linking, which coats the polyurea on the SAM. Polymer cross-linking enhances the robustness of the framework of the RSAM effectively. The crushing force increased from 0.4 N for the HSAM to 19.2 N for the RSAM. The pore volumes of HSAM (6.44 cm^3^/g) and RSAM (3.17 cm^3^/g) are much larger than their state-of-the-art counterparts. The specific surface areas of the HSAM and RSAM are 652 and 296 m^2^/g, respectively, which are at a high level. The organics’ sorption capacities of the RSAM are lower than those of the HSAM, owing to the poorer pore volume and specific surface area. However, the cyclic stability of the RSAM is better, benefiting from the reinforced network. The pump oil, hexane, and dilute MO sorption capacities of the HSAM are 17.9 g/g, 11.8 g/g, and 22.2 mg/g, respectively. The new SAM preparation method is facile, cost-effective, safe, and eco-friendly, and the resulting SAM sorbents are exceptional in capacity, dynamics, regenerability, and stability.

## 4. Materials and Methods

### 4.1. Materials

Aqueous sodium silicate (ASS, 33 wt%, modulus 3.3) was supplied by the Quechen Silicon Chemical Co., Ltd., Wuxi, China. Dimethicone oil (viscosity 1000 ± 80 mPa·s), aminopropyltriethoxysilane (APTES, 98%), hexamethyldisilazane (HMDS, 99%), and hexamethylene diisocyanate (HDI, 99%) were purchased from the Shanghai Aladdin Biochemical Technology Co., Ltd., Shanghai, China. Acetic acid (HAc, 99.5%) and acetonitrile (ACN, 99%) were purchased from the Sinopharm Chemical Reagent Co., Ltd., Shanghai, China. Ethanol (EtOH, 99.7%) was purchased from the Wuxi Yasheng Chemical Co., Ltd., Wuxi, China. Deionized water (W) was homemade in the laboratory. All reagents were used without any further purification.

### 4.2. Synthesis of the PSAM

The silica aerogel microspheres (SGMs) were synthesized through a continuous globing process on a self-built apparatus presented in Figure 1a. The inner and outer diameter of the silicone tube was 1.8 and 3.0 mm, respectively. The ASS and HAc solutions were prepared in advance by mixing ASS and HAc with water. The volume ratio of ASS/W was 6:14.1, while that of HAc/W was 1.2:18.9. Two solutions were mixed together with a peristaltic pump and an ultrasonic cleaner, and then auto-dropped into the oil bath of dimethicone at 90 °C to form the SGM. The SGM was washed with hot water three times to remove the external oil, then immersed in ethanol for solvent exchange at 50 °C for 48 h; the ethanol was changed four times to ensure all the water in SGM was replaced by ethanol thoroughly. The solvent-exchanged SGM was dried with supercritical CO_2_ to achieve the PSAM as control.

### 4.3. Synthesis of the HSAM

To obtain the HSAM, the ethanol-exchanged SGM was immersed in a HMDS/EtOH solution with a volume ratio of 2:1 for hydrophobic modification at room temperature for 48 h; the volume ratio of the modified solution to the SGM was 2:1. Subsequently, the modified SGM was washed with ethanol 3 times for 18 h to remove unreacted HMDS. Finally, the HMDS-modified SGM was dried with supercritical CO_2_ drying to obtain HSAM.

### 4.4. Synthesis of the RSAM

To obtain the RSAM, the ethanol-exchanged SGM was immersed in an APTES/EtOH/W solution with a molar ratio of 1:24:3 for amine grafting at 50 °C for 72 h. The amine-grafted SGM was washed with ACN at 50 °C, 4 times, within 48 h. Then, the amine-grafted SGM was immersed in an HDI/ACN solution at 50 °C for 48 h to obtain the reinforced SGM. The volume ratio of HDI/ACN was 1:1, and the volume ratio of the modified solution to the SGM was 2:1. The reinforced SGM was washed with ethanol at 50 °C, 4 times, within 48 h, whereafter the reinforced SGM was dried with supercritical CO_2_ to obtain the RSAM.

### 4.5. Characterization

The diameter of SAMs was obtained by selecting 100 microspheres from each PSAM, HSAM and PSAM and measuring them with vernier calipers and taking the average value. The apparent density was calculated from the weight and volume of the SAMs. For each sample, five microspheres were selected randomly to measure the average weight and volume. Fourier transform infrared spectroscopy (FTIR) was characterized on an IR-Spirit Fourier transform infrared spectrometer (Shimadzu, Kyoto, Japan) with ATR kit. Thermogravimetry/differential thermal analysis (TG/DTA) was performed on an HCT-1 thermogravimetric analyzer (HENVEN, Beijing, China) from 25 to 800 °C at a rate of 10 °C/min. The air flow rate was maintained at 30 mL/min. The water contact angle was measured on a JC2000D1 contact angle instrument (Zhongchen, Shanghai, China). SEM images and mapping were taken on a JEOL JSM-7600F field emission scanning electron microscope. TEM images were collected on a FEI Talos F200X G2 transmission electron microscope. A N_2_ adsorption/desorption test was performed on a Belsorp mini-X porosimeter. The mechanical performance was carried out on a DECCA-1 universal testing machine (Deka, Shenzhen, China). The organic absorption performances were evaluated with methyl orange (MO), hexane, and vacuum pump oil. The MO adsorption capacity was measured on a 722S UV/Vis spectrophotometer (Jinghua, Shanghai, China). The hexane and pump oil absorption capacities were evaluated by gravimetric method.

## Figures and Tables

**Table 1:** Characterization data of the PSAM, HSAM, and RSAM.

Sample	Apparent Density (g/cm^3^)	Specific Surface Area (m^2^/g)	Pore Volume (cm^3^/g)
SAM	0.080 ± 0.003	726	2.27
HSAM	0.108 ± 0.002	652	6.44
RSAM	0.166 ± 0.002	296	3.17

**Table 2:** Saturated oil absorption capacity of the HSAM and RSAM.

Sample	Pump Oil (g/g)	Hexane (g/g)
HSAM	17.9	11.8
RSAM	6.0	4.8

**Table 3:** Comparison of the specific surface area of SAMs and other adsorption materials.

Materials	SSA (m^2^/g)	Density (g/cm^3^)	Absorbance (g/g)	Refs.
SAMs	296–726	0.08–0.166	6.03–17.9	This work
Hydrophobic SiO_2_ aerogel	789	2.49	8.5	[13]
Cellulose aerogel	180	0.02–0.2	12.8	[50,51]
Activated carbon	500	0.45–0.65	-	[52,53]
Activated alumina	300	1.06	8	[54,55]
Polyurethane	655	0.4	17.7	[56]

## Data Availability

Not applicable.

## Supplementary Materials

The following supporting information can be downloaded at: https://www.mdpi.com/article/10.3390/gels8120778/s1, Figure S1: SEM mapping of the RSAM of C, N, O, Si element; Figure S2: Water contact angles of the (a) HSAM and (b) RSAM; Figure S3: Hydrophobicity and hydrophilicity of the HSAM treated at different temperatures ranging from room temperature to 450 °C; Figure S4: TEM images of (a) PSAM and (b) RSAM; Figure S5: Photograph of the force-stress test; Figure S6: Flow chart of the oil absorption with SAMs; Figure S7: Demonstration of the MO adsorption of RSAM; Figure S8: Diameter distribution of SAMs. (a) PSAM (b) HSAM (c) RSAM; Video S1: The auto-continuous synthesis of the SAMs; Video S2: The PSAM destroyed in water; Video S3: The RSAM retained in water.
