# Supplementary material for: Auto-Continuous Synthesis of Robust and Hydrophobic Silica Aerogel Microspheres from Low-Cost Aqueous Sodium Silicate for Fast Dynamic Organics Removal
Source: Gels. 2022 Nov 28;8(12):778. doi: 10.3390/gels8120778 (PMC9777793; doi:10.3390/gels8120778)
Supplement: Supplementary file 1 [file gels-08-00778-s001.zip › Supplementary Material-figures.pdf]

Supplementary Material for

Auto-continuous synthesis of robust and hydrophobic silica aerogel microspheres from low-cost aqueous sodium silicate for fast dynamic organics removal

Ziqian Sun [a,1], Zhiyang Zhao [a,1], Yong Kong [a, b, *], Jian Ren [a], Xing Jiang [a], Xiaodong Shen [a, b, *]

[a] College of Materials Science and Engineering, Nanjing Tech University, Nanjing 210009, China

[b] State Key Laboratory of Materials-Oriented Chemical Engineering, Nanjing 210009, China

* To whom correspondence should be addressed. E-mail: ykong@njtech.edu.cn (Y. Kong); xdshen@njtech.edu.cn (X. Shen)

[1] The authors contributed equally to this paper.

1 Supplementary figures

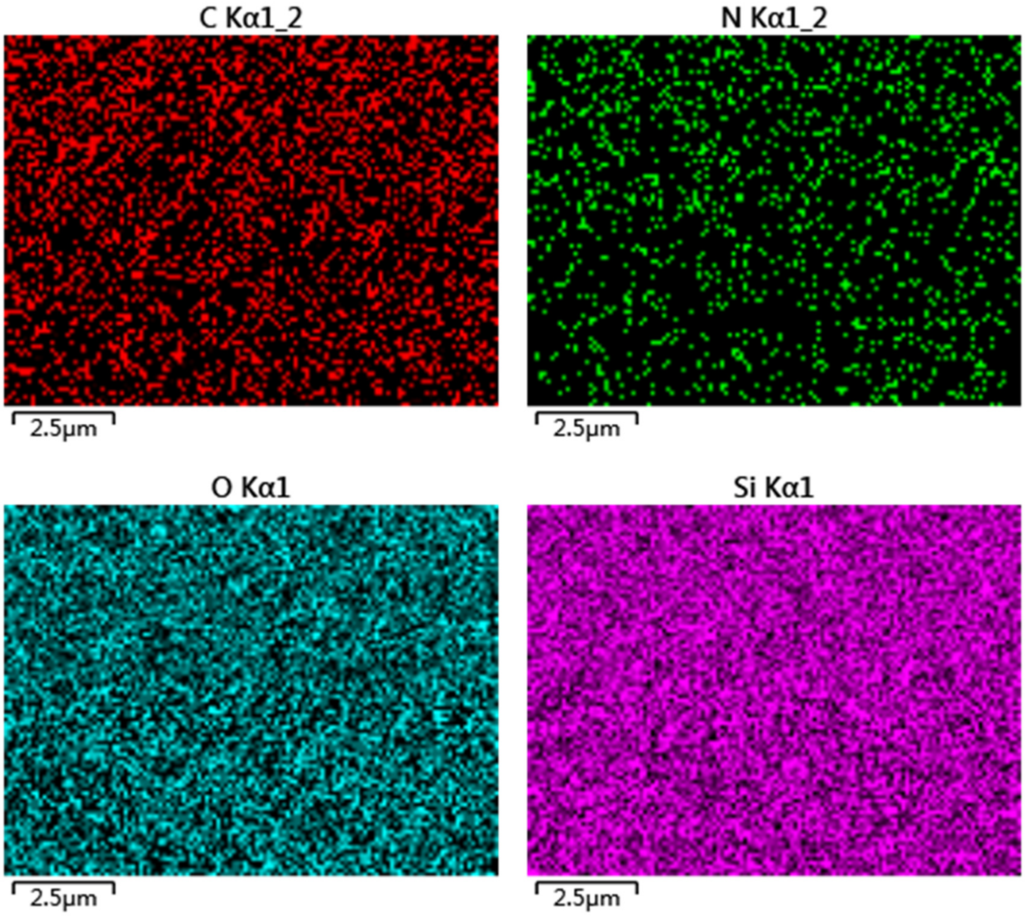

Figure S1. SEM mapping of the RSAM of C, N, O, Si element.

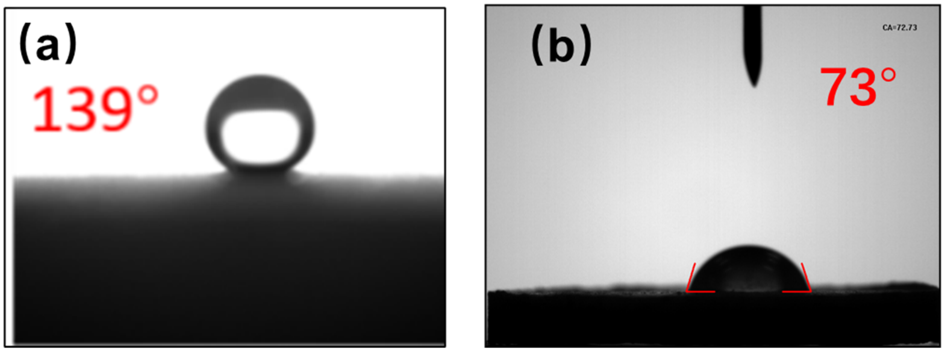

Figure S2. Water contact angles of the (**a**) HSAM and (**b**) RSAM.

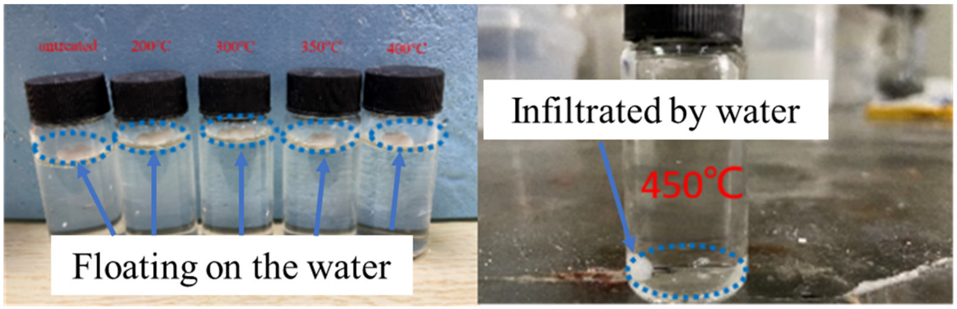

Figure S3. Hydrophobicity and hydrophilicity of the HSAM treated at different temperatures ranging from room temperature to 450 °C.

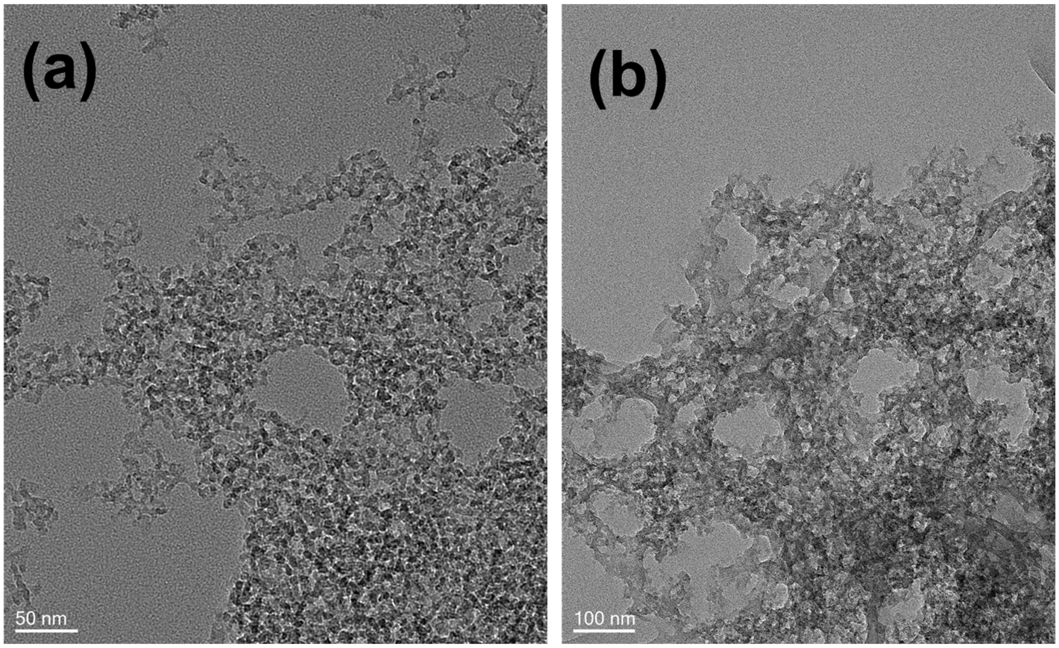

Figure S4. TEM images of (a) PSAM and (b) RSAM.

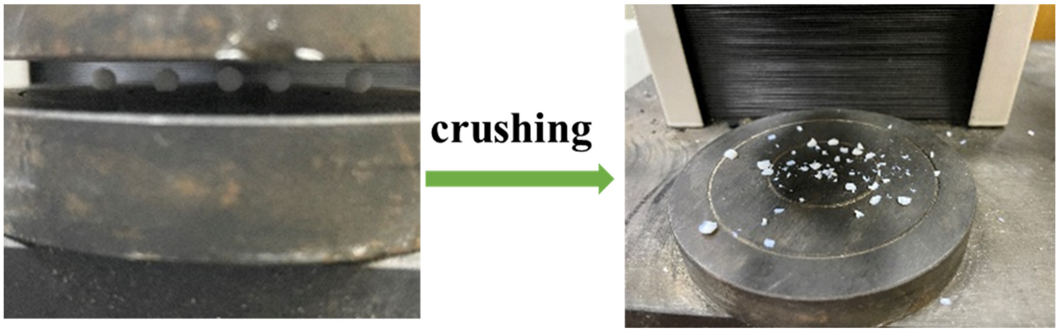

Figure S5. Photograph of the force-stress test.

2 Oil absorption test

The oil absorption capacity was measured by gravimetric method. As shown in Fig. S6, m_0 g of sorbent, HSAM or RSAM, was placed in a container with abundant oil, such as pump oil, sunflower seed oil and hexane. After 5 min, the saturating sorbent (m_1 g) was fished out. The oil absorption capacity was calculated by $(m_1-m_0)/m_0$. For pump oil absorption, the sorbent was regenerated by washing with ethanol and vacuum drying. For hexane absorption, the sorbent was regenerated by drying in a vacuum oven immediately. The weight of the regenerated sorbent (m_2 g) was measured to assess the regenerability of the sorbent, which was characterized by the desorption rate. The desorption rate was calculated by $(m_1-m_2)/(m_1-m_0)$. Cyclic absorption capacities were obtained by repeating the absorption-desorption processes.

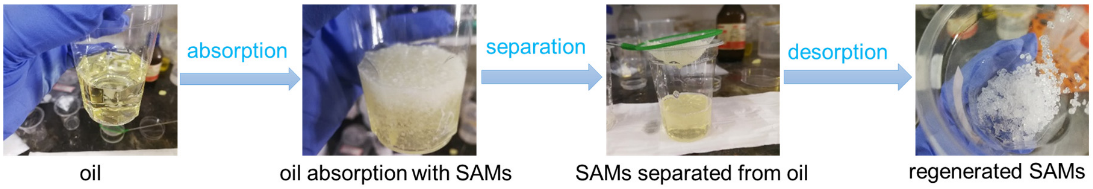

Figure S6. Flow chart of the oil absorption with SAMs.

3 MO adsorption test

To measure the MO adsorption capacity, the sorbent, HSAM or RSAM, was put in a MO solution (pH=7) with a concentration of 10 mg/L (C_0), as shown in Fig. S7. During oscillating, the MO concentration of the solution (C_e) was surveyed via spectrophotometer. The MO adsorption capacity (Q) of the SAMs can be calculated by the following equation:

$$Q = \frac{(C - Ce) \times V}{m}$$

where V represents the volume of the MO solution, m represents the weight of the sorbent.

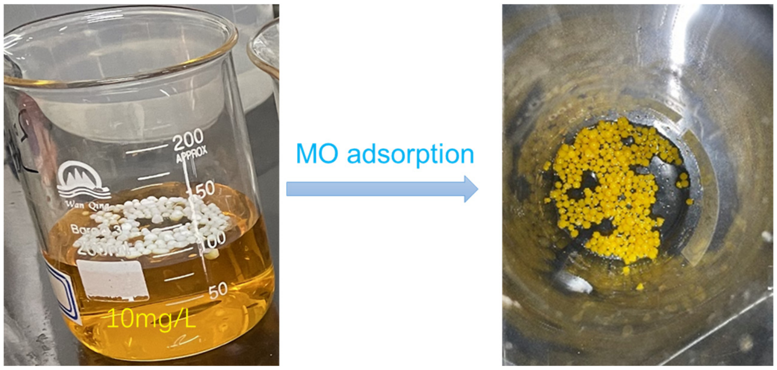

Figure S7. Demonstration of the MO adsorption of RSAM.

4 Diameter distribution test

To determine the particle diameter distribution of SAMs, 100 microspheres were randomly selected from each of PSAM, HSAM and PSAM and their diameters were measured using vernier calipers, the results of which are shown in Figure S8.

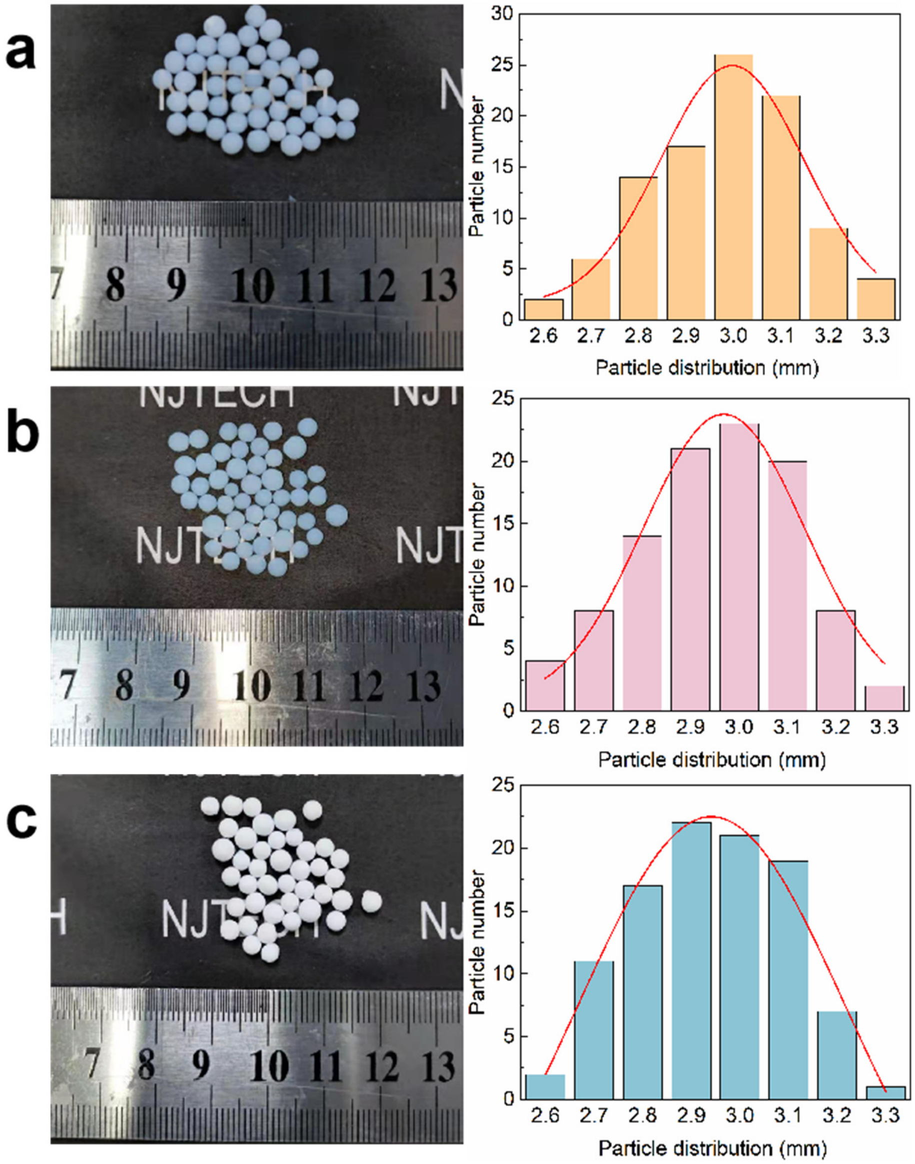

Figure S8. Diameter distribution of SAMs. (a)PSAM (b)HSAM (c)RSAM.
